# Association between tirofiban monotherapy and efficacy and safety in acute ischemic stroke

**DOI:** 10.1186/s12883-021-02268-8

**Published:** 2021-06-24

**Authors:** Chunrong Tao, Yuyou Zhu, Chao Zhang, Jianlong Song, Tianlong Liu, Xiaodong Yuan, Wenwu Luo, Changchun Chen, Dezhi Liu, Yuanyuan Zhu, Jie Liu, Wei Hu

**Affiliations:** 1 Stroke Center & Department of Neurology, Division of Life Sciences and Medicine, the First Affiliated Hospital of USTC, University of Science and Technology of China, Hefei, Anhui China; 2 Department of Pathology, The First Affiliated Hospital of Anhui Medical University, Hefei, Anhui China; 3 Department of Neurology, The Second People’s Hospital of Anhui Province, Hefei, Anhui China; 4 Department of Neurology, Shuguang Hospital Affiliated to Shanghai University of TCM, 528 Zhang-Heng Road,Pu-Dong New Area, Shanghai, 201203 China; 5 People’s Hospital of LiXin County, BoZhou City, 236700 AnHui Province China

**Keywords:** Acute ischemic stroke, Tirofiban, Modified Rankin scale, Mortality

## Abstract

**Background:**

Studies have suggested that glycoprotein IIb/IIIa antagonists such as tirofiban are beneficial for patients with acute coronary syndromes. However, it is still uncertain about the efficacy and safety of tirofiban in patients with acute ischemic stroke (AIS).

**Methods:**

In this prospective non-randomized study, 255 AIS patients were recruited from 4 comprehensive stroke centers in China between January, 2017 and May, 2018. Among them,169 patients were treated with aspirin plus clopidogrel and 86 patients were treated with tirofiban. The primary functional outcome was the distribution of the 90 days’ modified Rankin Scale (mRS). The safety outcomes included the incidence of intracranial hemorrhage (ICH) at discharge and mortality at 3 months.

**Results:**

In the propensity score matched cohort, tirofiban alone was noninferior to the dual antiplatelet with regard to the primary outcome (adjusted common odds ratio, 0.97; 95% confidence interval, 0.46 to 2.04; *P* = 0.93). Mortality at 90 days was 10% in the dual antiplatelet group and 8% in the tirofiban group (adjusted odds ratio 0.75; 95% CI 0.08 to 7.40, *p* = 0.81). There was no difference of the ICH rate between two groups (adjusted odds ratio 0.44; 95% CI 0.13 to 1.48, *p* = 0.18). In the inverse probability of treatment weighting-propensity score-adjusted cohort, similar differences were found for functional and safety outcomes.

**Conclusions:**

Our study suggested that tirofiban use appears to be safe as monotherapy in AIS treatment compared with common dual antiplatelet therapy, however, no improvement in functional outcomes was found.

**Trial registration:**

Chinese clinical trial registry, ChiCTR2000034443, 05/07/2020. Retrospectively registered.

## Background

Stroke is currently one of the leading causes of death and disability in China and all over the world [1]. Intravenous thrombolysis (IVT) with alteplase is the preferred therapy for patients with acute ischemic stroke (AIS) [2]. However, the treatment window of IVT is 4.5 h of symptoms onset, and only 2% of stroke patients could be beneficial from this treatment regimen [3, 4]. Other treatment options are therefore in demand for the AIS patients who could not receive IVT within the time window.

Antiplatelet therapy is the primary method to prevent and treat AIS [4, 5]. As a highly selective nonpeptide gpIIb/IIIa antagonist, tirofiban has been proven to be beneficial for the treatment of acute coronary syndrome [6]. However, the effect of tirofiban in AIS patients was still uncertain [7, 8]. The effect of platelet aggregation blockade of tirofiban is dose-dependent and can be rapidly reversible in 1.5 to 2.2 h. Therefore, compared with the oral antiplatelet drug clopidogrel and aspirin, intravenous tirofiban infusion is fast-acting and the short half-time makes it possible to swiftly repeal the antiplatelet effect. However, only few studies have evaluated the efficacy of tirofiban and some of them primarily focused on the efficacy of tirofiban in patients receiving IVT or endovascular treatment (EVT) [9, 10].

Three studies evaluating the safety and efficacy of tirofiban monotherapy suggested that the functional outcomes of AIS patients could not be improved. Junghans et al. found that the proportion of recovery, stable deficit or slight deterioration were not different (*p* = 0.18) between tirofiban group (*n* = 18, progressive deteriorating AIS) and matched controls (*n* = 17, stable status AIS) [11]. Randomly assigned 150 AIS patients to tirofiban or aspirin, Torgano et al. found that the reduction of National Institutes of Health Stroke Scale (NIHSS) great than 4 points and the mRS at 3 months were not different between the both groups [12]. Siebler et al. conducted a randomized study with 260 AIS patients, which reported no statistically significant difference of the modified Rankin Scale (mRS) and Barthel Index at 5 months between the tirofiban group and the placebo controlled group [13]. However, mortality at 5 months was significantly lower in tirofiban group (OR, 4.05; 95% CI, 1.1 to 14.9, *p* = 0.03). To date, the effect of tirofiban monotherapy is still controversial in terms of long-term function outcomes and mortality, and no study has yet evaluated the efficacy and safety of tirofiban monotherapy in the East Asian population or using dual antiplatelet as the control group.

Therefore, we present the data of a prospective multicenter trial on the efficacy and safety of tirofiban within the first 24 h of AIS.

## Methods

### Study design

This was a non-randomized, interventional study, where consecutive patients with AIS treated with tirofiban monotherapy or aspirin plus clopidogrel were enrolled prospectively at 4 stroke centers in China between January 2017 and May 2018. The patients were assigned to either tirofiban or control group in ratio of 1:2. The choice to treatment was determined by the clinical experience of the clinicians and by the patient preference. The intervention group received intravenous continuous infusion of tirofiban while the control group received standard dual antiplatelet treatment (aspirin and clopidogrel).

The study protocol was approved by the medical ethics committee of the first affiliated hospital of USTC. Written informed consent was given by all patients or their relatives before participation.

### Eligibility criteria

The inclusion criteria were as follows: age ≥ 18 years; on admission within 24 h after symptom onset. The exclusion criteria were: known thrombocytopenia at presentation or a thrombocyte count of 100 × 10^9^/L or lower; had intracranial hemorrhage (ICH); had malignant edema; renal insufficiency (creatinine clearance rate < 30 mL/minutes); and hepatic dysfunction (serum alanine transaminase >twice the upper limit of the normal value, or serum aspartate transaminase >twice the upper limit of the normal value); pregnant women; subjects disabled before the recent stroke (mRS > 2); recent major bleedings, surgery, or trauma.

### Medication

In the tirofiban group, patients received body-weight adjusted intravenous tirofiban infusion at the dose of 0.4 μg/kg/min for 30 min followed by a continuous infusion of 0.1 μg/kg/min for 48 h. After the infusion of tirofiban, patients received clopidogrel (75 mg per day) plus aspirin (100 to 200 mg per day). In the antiplatelet group, patients received clopidogrel (75 mg per day) plus aspirin (100 to 200 mg per day) at the beginning of treatment.

### Brain imaging

After the first cerebral CT examination to exclude primary hemorrhage or findings unrelated to the diagnosis of acute ischemic stroke, a second equally standardized cerebral CT examination was performed 2 to 6 days after the completion of the study medication. Information about the extent of early cerebral ischemia on baseline was measured by the Alberta Stroke Program Early Computed Tomography Score (ASPECTS).

### Data collection and assessment

Baseline demographic and clinical information for all enrolled patients were recorded, including age, sex, premorbid mRS, admission NIHSS score, stoke etiology, presence of hypertension, diabetes mellitus, hyperlipidemia, atrial fibrillation, smoking history, time from stroke onset to hospital admission and time from hospital admission to the treatment.

Our primary outcome was the scores on the mRS assessed at 90 days (within a window of ±14 days). Ninety days after the acute event, functional outcome was assessed by boardcertified vascular neurologists during a routinely scheduled clinical visit or by a study nurse certified in administering the mRS during a standardized telephone interview if the patient was unable to attend. Secondary outcomes included favorable outcome (90-day mRS score 0–3), excellent outcome (90-day mRS score 0–2), 24-h NIHSS shift (defined as baseline NIHSS to 24-h NIHSS) and 7 day or discharge NIHSS shift (defined as baseline NIHSS to 7 day or discharge NIHSS). Safety outcomes included the all-causes mortality at 90 days and the occurrence of cerebral hemorrhage according to the ECASS II (European Collaborative Acute Stroke Study) classification [14]. Imaging criteria were evaluated by a local investigator of each center.

### Statistical analyses

Continuous variables were described as mean and SD, and categorical variables were presented as absolute frequencies. Baseline characteristics were described according to the administered treatment, and the absolute standardized difference (ASD) was used to assess the magnitude of the between-group differences. A standardized difference of 10% is equivalent to a phi coefficient of 0.05 (negligible correlation), therefore, an ASD of 0.10 or more indicates that covariates are imbalanced between groups [15–17]. We compared the outcomes between the 2 study groups after taking into account the potential confounding factors by using prespecified propensity score methods (PSM) [18].

The effects of the treatment were estimated by using propensity score matching as primary analysis and by using inverse probability weighted regression adjustment (IPWRA) model (probability weights was obtained to calculate the outcome-regression parameters that account for the missing-data problem arising from the fact that each subject is observed in only one of the potential outcomes) as a secondary analysis.

A multivariable probit regression model was used to calculate the propensity score, and using antithrombotic therapy as dependent variable and all the obtained variables related to the outcomes as covariates. Patients in the dual antiplatelet group were matched 1:1 to patients in the tirofiban group based on corresponding PSM, using the nearest neighbor matching algorithm with a caliper width of 0.2 of the propensity score. To evaluate bias reduction after PSM, absolute standardized differences were calculated again after PSM [19, 20].

In IPWRA model, between-group comparisons were done with a three-step approach. Firstly, the parameters of the treatment model and the inverse-probability weights were calculated. Secondly, fit weighted regression models of the outcome for each treatment level and obtain the treatment-specific predicted outcomes for each subject with the estimated inverse-probability weights. Thirdly, calculate the means of the treatment-specific predicted outcomes, and the contrasts of these averages provide the estimates of the average treatment effect on the treated [21].

Statistical testing was conducted at the 2-tailed level of 0.05. All analyses were performed using STATA version 14.2 and the significance level was set at 0.05.

### Data availability

Anonymized data will be shared by request from any qualified investigators.

## Results

Between January 2017 and May 2018, a total of 255 AIS patients were admitted at the participating centers and satisfied the inclusion criteria. Of these, 169 patients were treated with aspirin plus clopidogrel and 86 patients were treated with tirofiban. The primary outcome of the mRS score at 90 days was missing for 18 patients (12 patients in the control group and 6 patients in the intervention group), and data were not imputed.

Fifty matched pairs were found in the primary analysis. Table shows the baseline characteristics according to the 2 study groups before and after PMS. Before matching, smoking history, stroke etiology, ASPECTS score, admission SBP and admission NIHSS showed meaningful differences (ASD > 10%). ASD reduced significantly after PSM with a maximum ASD of 0 for smoking history, 5.6% for TOAST classification, 15.2% for ASPECT score, 12.2% for admission SBP and 2.7% for NIHSS score (Table 1).

**Table 1:** Baseline characteristics according to anti-platelet approach in AIS Patients before and after PSM

	Before Matching			After Matching		
	Dual antiplatelet (*n* = 169)	Tirofiban (*n* = 86)	ASD, %	Dual antiplatelet (*n* = 50)	Tirofiban (*n* = 50)	ASD, %
Age, y, mean (sd)	72.4 (10.1)	72.4 (10.3)	0.4	70.1 (11.2)	70.7 (10.9)	5.4
Female, no. (%)	72 (42.6)	40 (46.5)	7.9	23 (46)	22 (44)	4.0
Smoke ever, no. (%)	30 (17.8)	24 (27.9)	24.4	8 (16)	8 (16)	0
Premorbid mRS, mean (sd)	0.4 (0.7)	0.4 (0.7)	2.0	0.5 (0.8)	0.5 (0.8)	5.2
Stroke etiology						
Large artery atherosclerosis	133 (78.7)	74 (86.0)	22.10	42 (84)	41 (82)	5.6
Cardioembolism	3 (1.8)	2 (2.3)		1 (2)	1 (2)	
Other	33 (19.5)	10 (11.6)		7 (14)	8 (16)	
ASPECTS, mean (sd)	7.9 (2.4)	8.3 (1.8)	22.3	8.2 (1.9)	7.9 (2)	15.2
Admission SBP, mm Hg, mean (sd)	155.4 (28.6)	141.9 (23.5)	51.7	147 (27.9)	143.9 (23.3)	12.2
Prior use of IV thrombolysis, no. (%)	11 (6.5)	7 (8.1)	6.3	4 (8)	5 (10)	7.0
Endovascular treatment, no. (%)	28 (16.6)	13 (15.1)	4.0	7 (14)	8 (16)	5.6
NIHSS score prior to treatment, mean (sd)	8 (3.6)	11.7 (5.5)	80.0	10.1 (3.6)	10 (5.1)	2.7
Medical history						
Hypertension, no. (%)	137 (81.1)	18.7	18.7	38 (76)	38 (76)	0
Diabetes, no. (%)	25 (14.8)	10.2	10.2	7 (14)	8 (16)	5.6
Hyperlipidemia, no. (%)	11 (6.5)	14.2	14.2	2 (4)	4 (8)	16.9
Atrial fibrillation, no. (%)	19 (11.2)	4.8	4.8	4 (8)	5 (10)	7.0
Coronary disease	56 (33.1)	22.1	22.1	12 (24)	11 (22)	4.8
Workflow, median (IQR), min						
onset to admission, h, mean (sd)	7.5 (3.9)	6.1	6.1	8.1 (4.5)	8 (3.7)	2.4
onset to treatment, h, mean (sd)	9.8 (4.2)	13.8	13.8	10.7 (5)	10.3(4.7)	6.4

Abbreviations: *ASPECTS* Alberta Stroke Program Early CT Score, *IQR* interquartile range, *mRS* modified Rankin Scale, *ASD* absolute standardized difference, *IV* intravenous, *NIHSS* National Institutes of Health Stroke Scale, *SBP* systolic blood pressure, *EVT* endovascular treatment

### Efficacy

**Fig. 1:**
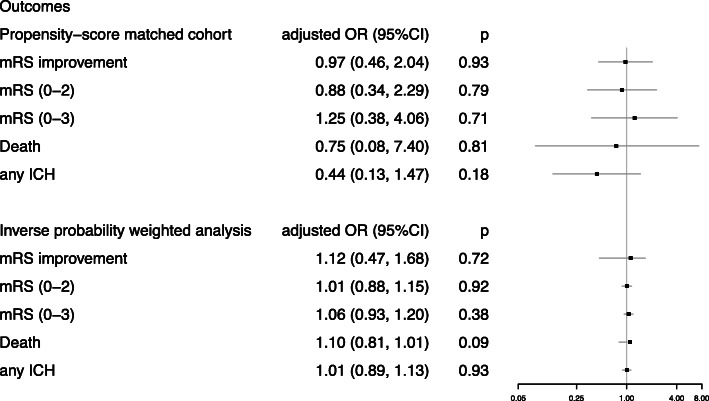
Comparisons in mRS score, mortality and ICH outcomes according to treatment allocation in AIS patients in propensity-score matched and inverse probability weighted analyses. Abbreviations: CI = confidence interval; OR = odds ratio; mRS = modified Rankin Scale. All regression analyses were adjusted for the following variables: age, sex, smoking history, baseline Alberta Stroke Program Early CT Score, baseline NIH Stroke Scale score, IV thrombolysis, endovascular treatment, premorbid mRS, TOAST classification, hypertension, diabetes mellitus, hyperlipidemia, atrial fibrillation, coronary disease, onset to admission and onset to treatment

**Fig. 2:**
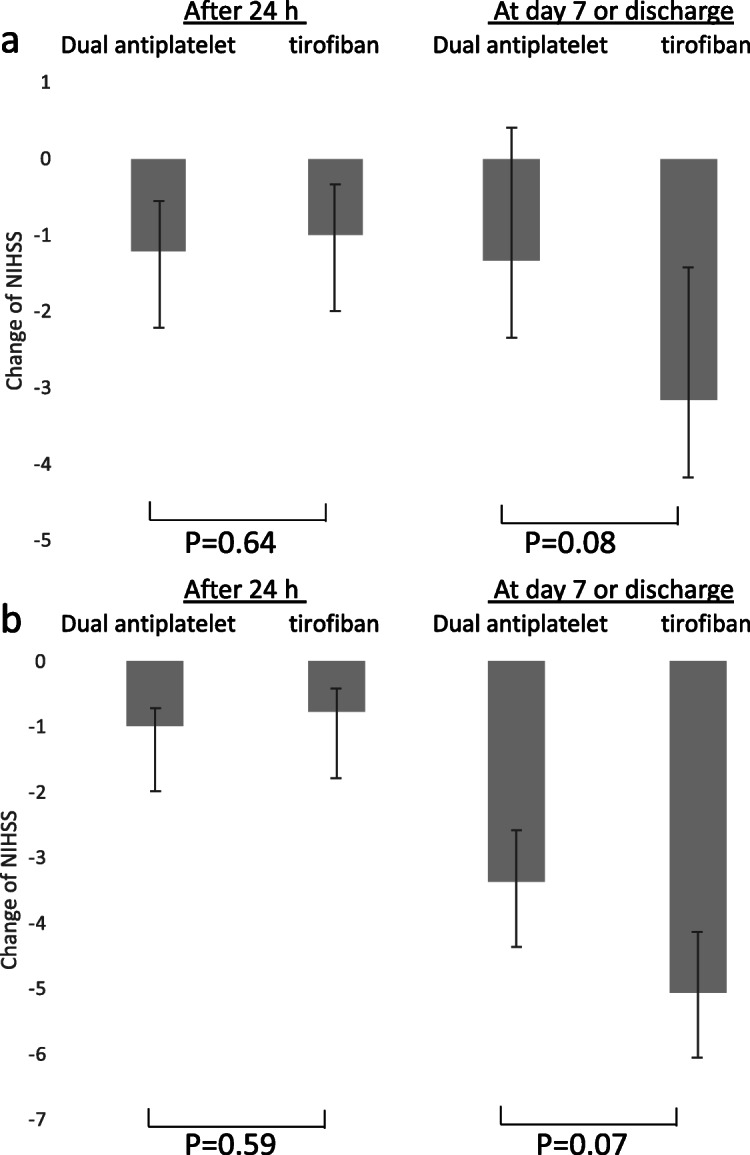
Association of treatment allocation with NIHSS change at after 24 h or at day 7 or hospital discharge in (**a**) propensity-score matched and (**b**) inverse probability weighted analyses. Abbreviation: NIHSS = National Institutes of Health stroke scale. All regression analyses were adjusted for the following variables: age, sex, smoking history, baseline Alberta Stroke Program Early CT Score, baseline NIH Stroke Scale score, IV thrombolysis, endovascular treatment, premorbid mRS, TOAST classification, hypertension, diabetes mellitus, hyperlipidemia, atrial fibrillation, coronary disease, onset to admission and onset to treatment

In the PSM cohort, the adjusted common odds ratio for the mRS score at 90 days was 0.97 (95% confidence interval [CI], 0.46 to 2.04; *P* = 0.93) (Fig. 1a). Favorable outcome was not different between the control group (32%) and the intervention group (34%, matched odds ratio = 1.25, 95% CI, 0.38 to 4.06, *p* = 0.71). As shown in Fig. 2a, the improvement of NIHSS after 24 h showed no significant difference between the two groups (*p* = 0.64): − 1.22 for the dual antiplatelet group and − 1.00 for the tirofiban group, respectively. No association was seen between treatment allocation and improvement in NIHSS at 7 days as well (*p* = 0.08). In IPWRA model, similar results were found for efficacy outcomes (Figs. 1 and 2b).

### Safety

In the PSM cohort (Fig. 1a), mortality at 90 days was 8.0% in the control group and 10.0% in the intervention group (adjusted odds ratio 0.75. 95% CI 0.08 to 7.40, *p* = 0.81). There was no difference (*p* = 0.18) of the percentages of patients with ICH in the dual antiplatelet group (24% [12 patients]) and in the tirofiban monotherapy group (8% [16 patients]). The sensitivity analysis with IPWRA model provided similar results (Fig. 1b).

## Discussion

Our study was designed to test the efficacy and safety of tirofiban in patients with AIS. We found that tirofiban use was not associated with mRS score at 3-month, and the risk of ICH as well as the mortality were not increased compared with the standard dual antiplatelet care.

The optimal antithrombotic approach during AIS is still uncertain. Patients with the administration of tirofiban showed no improvement in functional outcome as measured by mRS at 3 months, which was in agreement with the previous studies evaluating the efficacy and safety of tirofiban monotherapy in non-selected AIS population [11–13, 22]. In AIS patients receiving EVT, studies have also demonstrated that the use of tirofiban could not increase the proportion of patients with mRS 0–2 at 3 months [9, 10, 23–30]. Studies failed to reach a consensus about whether the efficacy of tirofiban was superior in patients receiving IVT with alteplase [31, 32].

Our study also found that the reduction of NIHSS at 24 h and at 7 days or discharge were similar between two groups. Torgano et al. found that the reduction of NIHSS ≥4 was seen in 56% of cases in both tirofiban group and aspirin group [12]. Including 82 patients, Li et al. found that NIHSS score at 7 days was significantly lower with alteplase plus tirofiban as compared to alteplase alone (*p* = 0.002) [5]. The association between tirofiban monotherapy and the early neurological functional change could not be determined yet, and the future prospective cohort study is needed to clarify this association.

There was no significant difference in the incidence of ICH between the two groups. Several studies have also demonstrated that the combination of tirofiban and other standard cares did not increase the risk of ICH [13, 31, 33]. It can be inferred that the administration of tirofiban instead of dual antiplatelet is relatively safe in AIS patients who had fulfilled the inclusion criteria.

Our study found that the mortality was similar between the control group and the tirofiban group after the 3 months’ follow-up. In line with our results, one recent meta-analysis demonstrated that the mortality at 3 months did not increase after the administration of tirofiban (OR, 0.80; 95% CI; 0.64–1.02; *p* = 0.07) [22]. Wu et.al performed a multicenter retrospective cohort study including 187 Chinese patients found that tirofiban use was associated with an insignificant but lower mortality at 3 months after IV thrombolysis (OR, 0.77; 95% CI, 0.19–2.27; *P* = 0.875) [32].

There are several limitations of this study. First, despite that we used propensity score analysis to minimize the difference in baseline characteristics, our results could be confounded by variables that were not included in the propensity model. Second, when evaluating the long-term functional outcome, adjunct therapies administered to the patients in the first 3 months following stroke were not controlled for and may have influenced the outcome. Third, relatively small sample size prevented us from performing further stratification analysis in the PMS cohort.

## Conclusion

Through a more specifically and reversibly binding to platelet GP IIb/IIIa receptors, and a shorter half-life of 2 h, tirofiban use was associated with a similar rate of hemorrhagic complications whether it is administered alone or following IVT or EVT. An acceptable efficacy and safety profile allowed for alternative or adjuvant treatment to current management of early AIS. For AIS patients at high risk of refractoriness or progression, the use of tirofiban have been providing a new perspective for the validated treatment. Its inhibition effect on ongoing platelet aggregation and thrombosis, rather than absolute thrombolytic effect suggests that tirofiban may be promising in future AIS treatment.

To the best of our knowledge, this is the first study evaluating the association between tirofiban monotherapy within the 24 h after symptom onset and prognosis in AIS patients in the East Asian population. Our results indicate that tirofiban could not improve functional outcomes compared to the standard dual antiplatelet care in AIS patients, but the low ICH rate and mortality suggested that it is safe when administered early as monotherapy. Further prospective cohort studies with large sample size are required to provide robust evidence.

## Data Availability

The datasets used and/or analysed during the current study available from the corresponding authors (WH and JL) on reasonable request.

## Abbreviations

AIS: Acute ischemic stroke
mRS: Modified Rankin Scale
ICH: Intracranial hemorrhage
NIHSS: National Institutes of Health Stroke Scale
ASPECTS: Alberta Stroke Program Early Computed Tomography Score
ASD: Absolute standardized difference
PSM: Propensity score methods
IPWRA: Inverse probability weighted regression adjustment
OR: Odds ratio
